# Development of an indirect ELISA for detection of anti-*Mycoplasma hyopneumoniae* IgG in naturally infected pathogen-induced convalescent sera

**DOI:** 10.1186/s12917-021-02828-7

**Published:** 2021-03-16

**Authors:** Yaqin Tian, Zuobo Xu, Yukang Wen, Mei Yang, Yaru Ning, Zhaodi Wang, Honglei Ding

**Affiliations:** 1 Laboratory of Veterinary Mycoplasmology, College of Veterinary Medicine, Southwest University, 2 Tiansheng Road, Beibei District, 400715 Chongqing, China; 2 Immunology Research Center, Medical Research Institute, Southwest University, 2 Tiansheng Road, Beibei District, 400715 Chongqing, China

**Keywords:** *Mycoplasma hyopneumoniae*, Indirect ELISA, Convalescent sera, IgG, Mhp366

## Abstract

**Background:**

Immunization of pigs with an inactivated *Mycoplasma hyopneumoniae* vaccine (bacterin) generates hyperimmune serum that contains high concentrations of anti-*M. hyopneumoniae* IgG. Commercially available IgG-ELISA kits cannot distinguish between anti-*M. hyopneumoniae* IgG in inactivated bacterin-induced hyperimmune sera and convalescent sera resulting from natural *M. hyopneumoniae* infection. Establishment of an ELISA to detect anti-*M. hyopneumoniae* IgG in convalescent sera will facilitate the evaluation of the *M. hyopneumoniae* status of pig farms.

**Results:**

In this study, we expressed and purified recombinant Mhp366-N protein, which contains an epitope recognized by *M. hyopneumoniae* convalescent sera but not hyperimmune sera, for use as a coating antigen. For the *M. hyopneumoniae* convalescent serum IgG-ELISA, the optimal antigen concentration, blocking buffer, blocking time, dilution of serum, incubation time with serum, secondary antibody dilution, secondary antibody incubation time and colorimetric reaction time were 0.25 µg/mL, 2.5 % skim milk, 1 h, 1:500, 0.5 h, 1:10,000, 1 h and 15 min, respectively. Validation of the *M. hyopneumoniae* convalescent serum IgG-ELISA showed a cut-off value of 0.323, the intra-assay CV ranged from 3.27 to 7.26 %, the inter-assay CV ranged from 3.46 to 5.93 %, and the assay was able to differentiate convalescent sera from antibodies to 7 other porcine respiratory pathogens. The convalescent serum IgG-ELISA detected no anti-*M. hyopneumoniae* IgG in hyperimmune serum samples while a commercial IgG-ELISA identified 95/145 of these sera as positive. The accuracy of the *M. hyopneumoniae* convalescent serum IgG-ELISA was comparable to the sIgA-ELISA but better than the commercial IgG-ELISA.

**Conclusions:**

The convalescent serum IgG-ELISA is a reproducible, sensitive, and specific indirect ELISA to detect anti-*M. hyopneumoniae* IgG in naturally infected pathogen-induced convalescent sera. This ELISA could be used to carry out large scale surveillance of *M. hyopneumoniae* infection in pig farms regardless of vaccination status.

## Background

*Mycoplasma hyopneumoniae* is the causative agent of porcine enzootic pneumonia (PEP). PEP is a widespread chronic respiratory disease of swine that is characterized by coughing, reduced weight gain, and decreased feed conversion [1]. *M. hyopneumoniae* infection is restricted to the lung [2] and exhibits high morbidity and low mortality [1]; however, PEP continues to have a substantial economic impact on the swine industry, worldwide [3]. Some studies have reported that *M. hyopneumoniae* infection increases the susceptibility of swine to secondary infection, causing porcine respiratory disease complex (PRDC) [1, 4].

Diagnosis of *M. hyopneumoniae* infection may be achieved by isolation of the bacterium, molecular identification, and serological detection. However, each of these methods is associated with several limitations. *M. hyopneumoniae* culture is time-consuming and costly and frequently contaminated by *M. hyorhinis* and *M. flocculare*, despite the use of selective media [5]. Real-time polymerase chain reaction has been successfully applied to identify and differentiate *M. hyopneumoniae* from *M. hyorhinis* and *M. flocclare* in PEP-like lesions, but this method is too expensive for routine use in testing laboratories, and sample collection can be challenging [6, 7]. Serological detection can be performed using an indirect ELISA that detects anti-*M. hyopneumoniae* IgG, although this assay has low sensitivity during early infection [8]. A sIgA-ELISA was developed to detect natural *M. hyopneumoniae* infection rather than the secretory IgA (sIgA) antibody raised by inactivated *M. hyopneumoniae* vaccine (bacterin) [9, 10], but this ELISA requires collection of nasal swabs, which is laborious and only yields a small amount of sample. Consequently, there remains an unmet need for a more sensitive and convenient method to diagnose natural *M. hyopneumoniae* infection.

A previous study identified a single strongly immunoreactive epitope on the Mhp366 protein of *M. hyopneumoniae* that reacted with an antibody in the sera of naturally infected pigs, but not in pigs immunized with bacterin [11]. In addition, Mhp366 was not detected in total cell lysates of *in vitro* grown *M. hyopneumoniae* strains, using a polyclonal serum raised against Mhp366 [11]. Based on the characteristics of the Mhp366 protein, we developed two ELISAs, one for screening serological immunodominant protein antigens [12] and another for further screening the discriminative immunodominant proteins that can distinguish between anti-*M. hyopneumoniae* IgG in inactivated bacterin-induced hyperimmune sera and convalescent sera resulting from natural *M. hyopneumoniae* infection [13]. The studies identified 15 serological immunodominant proteins and 1 discriminative serological immunodominant protein, Mhp462 [14]. These data suggest that a Mhp366-based ELISA has potential to be used as a diagnostic tool to detect natural *M. hyopneumoniae* infection.

The objective of this study was to develop an indirect ELISA (the *M. hyopneumoniae* convalescent serum IgG-ELISA) for the detection of a *M. hyopneumoniae* systemic serological IgG induced by natural infection, but not bacterin immunization, with higher sensitivity than the currently available commercial IgG-ELISA. The new ELISA should provide a precise method for evaluating the *M. hyopneumoniae* status in pig farms.

## Results

### Expression and purification of Mhp366-N

**Fig. 1:**
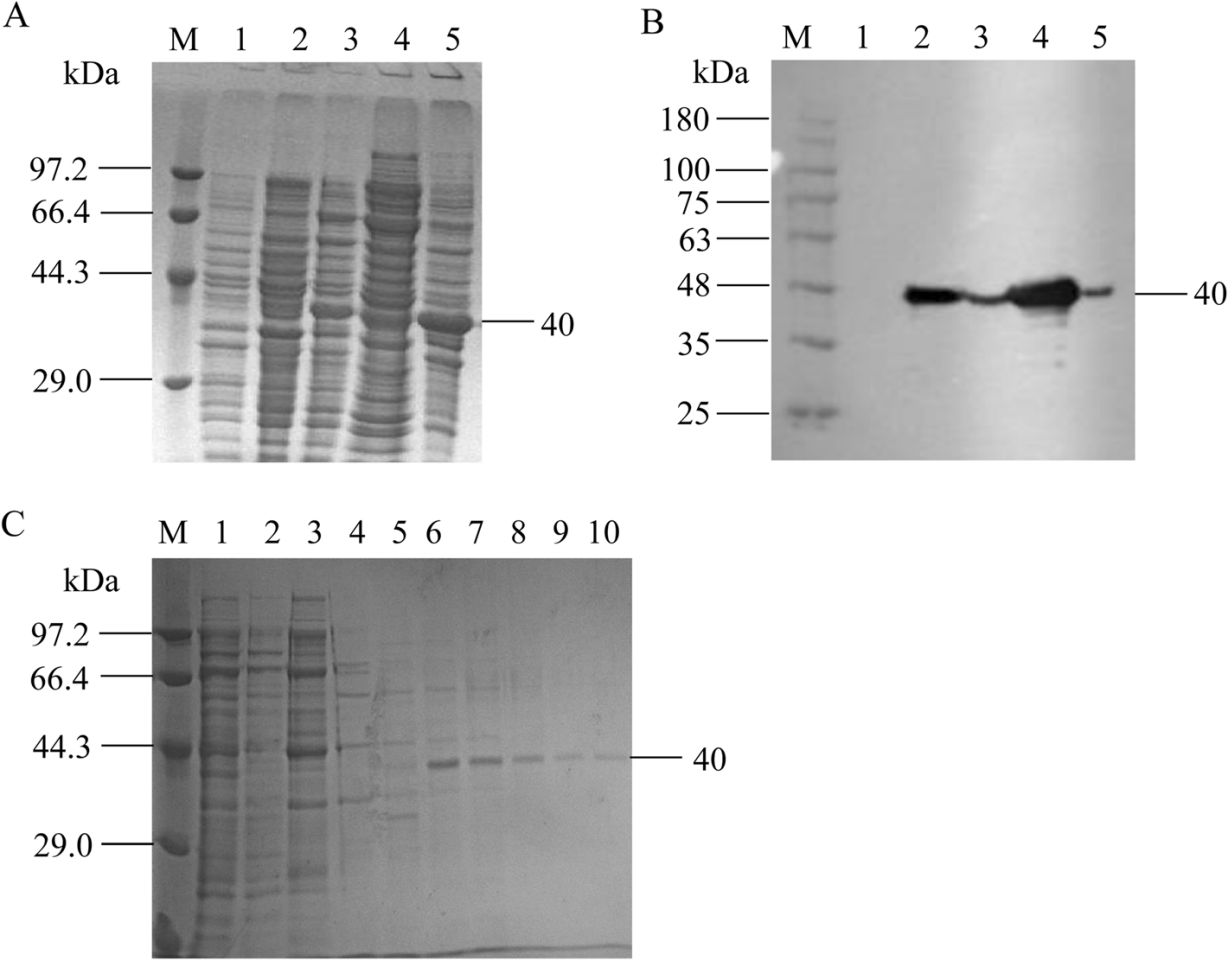
Expression and purification of the Mhp366-N protein. SDS-PAGE (**a**) and Western blotting (**b**) showing Mhp366-N in recombinant bacteria. Soluble (Lane 2, Fig. 1 **a** and **b**) and insoluble forms (Lane 3, Fig. 1 **a** and **b**) of Mhp366-N protein were expressed using IPTG induction (Lane 1, Fig. 1 **a**). No Mhp366-N was detected in *E. coli* BL21(DE3) containing the pET-28a(+) empty vector (Lane 1, Fig. 1 **b**). **c** Mhp366-N protein was purified by Ni affinity chromatography. Lane 1: loading material; Lane 2: flow through; Lane 3–5: affinity purification with a linear imidazole gradient, 0.1 M (Lane 3), 0.2 M (Lane 4), and 0.5 M (Lane 5); Lane 6–9: isolation of target protein

The 1 to 837 nucleotide sequence of *mhp366* was cloned into the expression vector pET-28a(+) and expressed in *E. coli* BL21(DE3). Soluble and insoluble forms of Mhp366-N protein were expressed, yielding a 40 kDa protein band on SDS-PAGE (Fig. 1a). These findings were confirmed on Western blot analysis using the His-tag antibody (Fig. 1b). The soluble recombinant 6×His-tagged protein was purified using Ni chelating affinity chromatography (Fig. 1c).

### Sample collection

Prevalence of antibodies to *M. hyopneumoniae* and *M. hyopneumoniae* DNA in sera and laryngeal swabs from Farm A and Farm B are summarized in Table 1. One hundred and eighty serum samples collected from unvaccinated pigs at Farm A were negative for *M. hyopneumoniae* IgG by commercial IgG-ELISA and the *P36* gene was not detected in the corresponding laryngeal swabs by nested PCR. Among the samples collected from Farm B, the prevalence of anti-*M. hyopneumoniae* IgG was 60 % (12/20), the *P36* gene was detected in nine laryngeal swabs by nested PCR, and seven samples were confirmed *M. hyopneumoniae* positive and six samples were confirmed *M. hyopneumoniae* negative on both ELISA and nested PCR. Finally, 180 *M. hyopneumoniae* negative sera from Farm A and 5 *M. hyopneumoniae* convalescent sera, collected from pigs that were positive by PCR testing, from Farm B were randomly selected for use in the following assay.

**Table 1:** Prevalence of *M. hyopneumoniae* DNA and IgG in pigs from Farm A and Farm B

Farm	No. of pigs	PCR for LS		Commercial ELISA for sera	
		+	-	+	-
A	180	0	180	0	180
B	20	9	11	12	8

*LS* laryngeal swabs

+, positive; -, negative

### Optimization of the working conditions of the *M. hyopneumoniae* convalescent serum IgG-ELISA

**Fig. 2:**
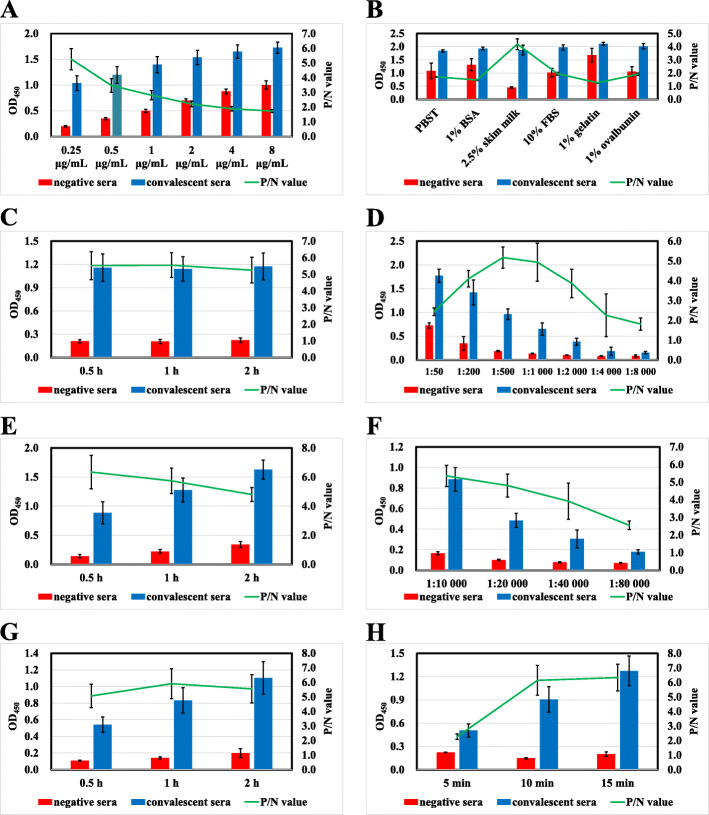
Optimization of the working conditions of the *M. hyopneumoniae* convalescent serum IgG-ELISA. Optimal coating antigen concentration was 0.25 µg/mL in coating buffer (**a**). The optimal blocking buffer was 2.5 % skim milk dissolved in PBS (**b**), and the optimal incubation time for the blocking step was 1 h (**c**). The optimal dilution of serum and secondary antibody were 1:500 (**d**) and 1:10,000 (**f**) diluted in blocking buffer. The optimal incubation times for serum and secondary antibody were 0.5 h (**e**) and 1 h (**g**). The optimal colorimetric reaction time was 15 min (**h**)

Optimization of the working conditions of the *M. hyopneumoniae* convalescent serum IgG-ELISA involved varying the concentration of antigen, the blocking buffer and incubation time, and the dilutions of serum and secondary antibody. Optimal coating antigen concentration was 0.25 µg/mL (Fig. 2a). 2.5 % skimmed milk dissolved in PBS was identified as the most efficient blocking agent (Fig. 2b), and the optimal incubation time for the blocking step was 1 h (Fig. 2c). Optimal dilution of *M. hyopneumoniae* convalescent and negative sera was 1:500 (Fig. 2d), and the optimal incubation time with sample was 0.5 h (Fig. 2e). Optimal dilution of HRP-conjugated rabbit anti-pig IgG (H + L) secondary antibody was 1:10,000 (Fig. 2f), and the optimal incubation time with secondary antibody was 1 h (Fig. 2g). The optimal colorimetric reaction time was 15 min (Fig. 2h).

### Cut-off value for the *M. hyopneumoniae* convalescent serum IgG-ELISA

The average OD_450_ value of 180 negative sera on the *M. hyopneumoniae* convalescent serum IgG-ELISA was 0.2, and the SD was 0.041, resulting in a cut-off value of 0.323 (mean ELISA value + 3SD = 0.323). Therefore, samples from unvaccinated pigs with OD_450_ values ≥ 0. 323 were considered *M. hyopneumoniae* convalescent sera, and samples from unvaccinated pigs with OD_450_ values < 0. 323 were considered negative.

### Reproducibility, specificity and sensitivity of the *M. hyopneumoniae* convalescent serum IgG-ELISA

Reproducibility of the *M. hyopneumoniae* convalescent serum IgG-ELISA was assessed by determining the intra- and inter-assay variation. The intra-assay CV of 2 negative serum samples and 2 convalescent serum samples ranged from 3.27 to 7.26 %, and the inter-assay CV of these samples ranged from 3.46 to 5.93 %, suggesting the ELISA is reproducible, yielding low and acceptable variations.

**Fig. 3:**
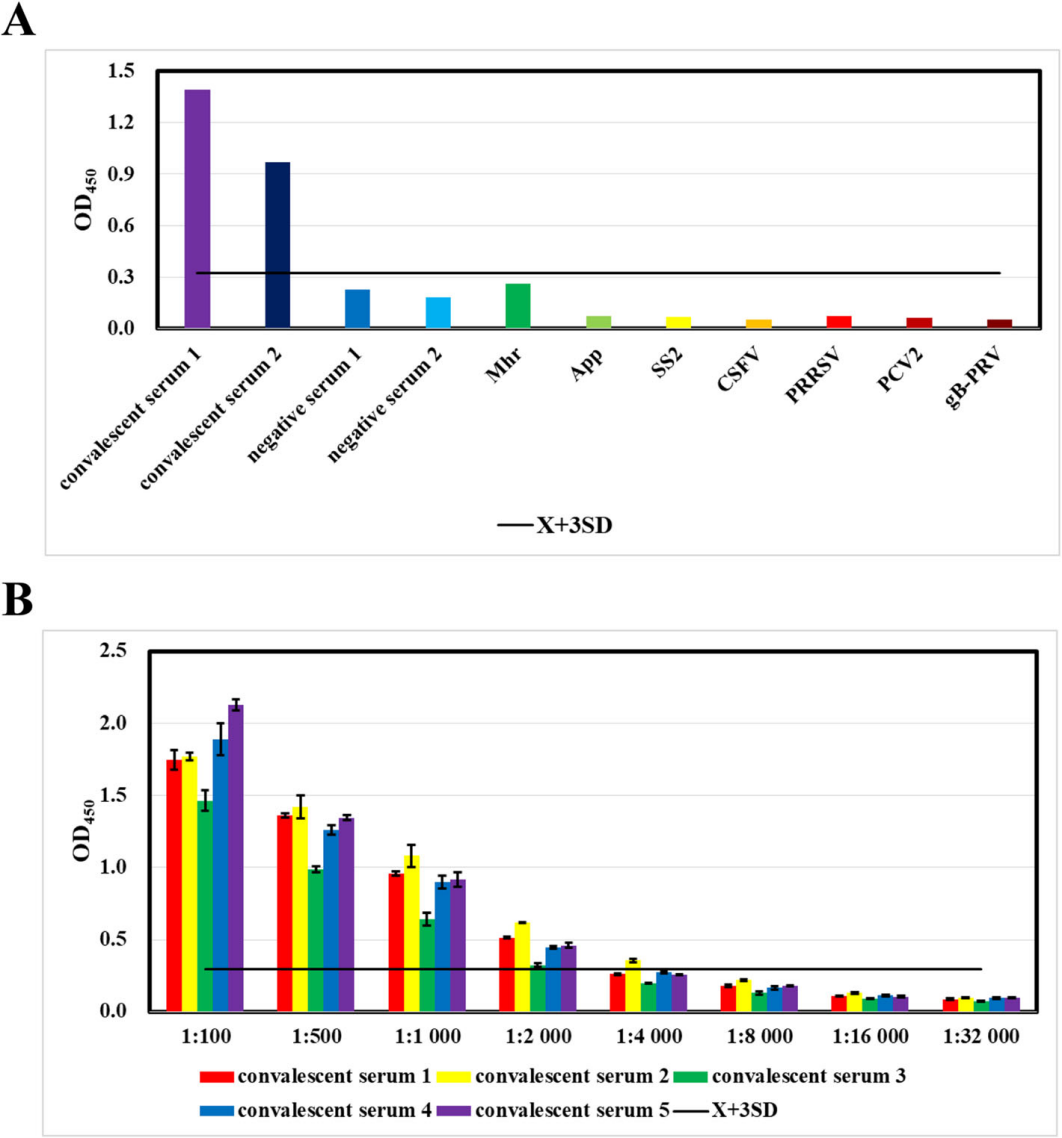
Specificity and sensitivity of the *M. hyopneumoniae* convalescent serum IgG-ELISA. **a** The ELISA detected no cross reactions with sera containing antibodies against 7 porcine respiratory pathogens, including *M. hyorhinis* (Mhr), *A. pleuropneumoniae* (App), *S. suis* serotype 2 (SS2), classical swine fever virus (CSFV), porcine reproductive and respiratory syndrome virus (PRRSV), porcine circovirus type 2 (PCV2) and pseudorabies virus gB protein (gB-PRV). **b** Five sera gave positive results at dilutions of 1:100, 1:500, 1:1,000 and 1:2,000, 1 serum gave a positive result at a dilution of 1:4,000, and 5 sera gave negative results at dilutions of 1:8,000 or more. Convalescent serum could be diluted up to 2,000 times for this assay

The ELISA detected no cross reactions with sera containing antibodies against 7 porcine respiratory pathogens, including Mhr, App, SS2, CSFV, PRRSV, PCV2 and gB-PRV (Fig. 3a). These data indicate the ELISA is specific for *M. hyopneumoniae* antibody induced by natural infection, and there was no cross reaction with sera containing antibodies against other swine respiratory pathogens.

The sensitivity of the ELISA was determined by diluting *M. hyopneumoniae* convalescent sera. Five sera gave positive results at dilutions of 1:100, 1:500, 1:1,000 and 1:2,000, 1 serum gave a positive result at a dilution of 1:4,000, and 5 sera gave negative results at dilutions of 1:8,000 or more (Fig. 3b), suggesting the ELISA provides sensitive detection of anti-*M. hyopneumoniae* IgG.

### Comparisons with different detection methods

Samples collected from Farm C were evaluated by 4 methods. Nested PCR was used to detect *M. hyopneumoniae* DNA in laryngeal samples, in which 19/80 samples were considered positive and 61/80 samples were considered negative. A commercial IgG-ELISA kit detected anti-*M. hyopneumoniae* IgG in 46/80 serum samples and the sIgA-ELISA kit detected *M. hyopneumoniae* IgA in 74/80 nasal swabs. The *M. hyopneumoniae* convalescent serum IgG-ELISA detected anti-*M. hyopneumoniae* IgG in 73/80 serum samples, and 7 samples were considered IgG negative (Table 2).

**Table 2:** Detection of *M. hyopneumoniae* DNA, IgG and sIgA by nested PCR, a commercial IgG-ELISA, the *M. hyopneumoniae* convalescent serum IgG-ELISA, and the sIgA-ELISA in laryngeal swabs, sera, and nasal swabs collected from pigs at farm C

Status	Nested PCR	Commercial IgG-ELISA	*M. hyopneumoniae* convalescent serum IgG-ELISA	SIgA-ELISA
Positive	19	46	73	74
Negative	61	22	7	6
Suspect	0	12	0	0

The sIgA-ELISA is currently applied as the most accurate method to detect natural *M. hyopneumoniae* infection [10]. Therefore, we compared the accuracy of a commercial IgG-ELISA kit, the convalescent serum IgG-ELISA and the sIgA-ELISA in sera and nasal swabs, respectively (Table 3). 96.3 % of the sera tested on both the sIgA-ELISA and convalescent serum IgG-ELISA gave consistent results, including 97.3 % of the sera that tested positive and 83.3 % of the sera that tested negative. Only 56.3 % of the sera tested on both the sIgA-ELISA and commercial IgG-ELISA gave consistent results, including 58.1 % of the sera that tested positive and 58.1 % of the sera that tested negatively.

**Table 3:** Comparison of a commercial IgG-ELISA, the *M. hyopneumoniae* convalescent serum IgG-ELISA, and the sIgA-ELISA using serum samples and nasal swabs collected from 80 pigs at Farm C

SIgA-ELISA	Commercial IgG-ELISA				*M. hyopneumoniae* convalescent serum IgG-ELISA		
	**Positive**	**Suspect**	**Negative**	**Total**	**Positive**	**Negative**	**Total**
Positive	43	11	20	74	72	2	74
Negative	3	1	2	6	1	5	6
Total	46	12	22	80	73	7	80

Theoretically, hyperimmune sera from vaccinated pigs should not be detected as positive using the *M. hyopneumoniae* convalescent serum IgG-ELISA. To verify this, sera from 145 pigs immunized with *M. hyopneumoniae* inactivated bacterin were evaluated with the IDEXX *M. hyopneumoniae* IgG-ELISA and the *M. hyopneumoniae* convalescent serum IgG-ELISA. The IDEXX *M. hyopneumoniae* IgG-ELISA identified 95/145 sera as positive and 50/145 as negative. In contrast, all sera were considered negative by the *M. hyopneumoniae* convalescent serum IgG-ELISA, indicating that our novel ELISA can distinguish between naturally infected pathogen-induced convalescent sera and inactivated bacterin-stimulated hyperimmune sera.

## Discussion

Infection with *M. hyopneumoniae* has important economic implications for the pig industry, causing decreased feed efficiency, reduced average daily weight gain, and increased medication costs [15]. PCR and ELISA are used to detect *M. hyopneumoniae* DNA and antibodies, respectively, but both methods are associated with several limitations. Real-time PCR testing on tracheo-bronchial swabs provides a sensitive method for confirming *M. hyopneumoniae* infection by detecting the *mhp183* gene [16]; however real-time PCR is more expensive than ELISA, traditional PCR may lack sensitivity compared to real-time PCR, and findings on PCR can be influenced by the sampling method, whereby analysis of nasal swabs, laryngeal swabs, tracheobronchial swabs, bronchoalveolar lavage fluid, or lung tissues by different operators may provide diametrically opposing results [6, 17–19]. With regard to serological detection, a blocking ELISA (IDEI, *M. hyopneumoniae* EIA kit, Oxoid) and two indirect ELISAs (HerdCheck, IDEXX and Tween 20-ELISA) can detect anti-*M. hyopneumoniae* IgG [20]; however, these assays cannot discriminate between pigs with natural *M. hyopneumoniae* infection and vaccinated pigs [9, 10]. The sIgA-ELISA provides a sensitive and accurate method to identify *M. hyopneumoniae* sIgA in nasal swabs. However, nasal swabs can only be stored at -20℃ for a short time. Based on our experience, sIgA loses its activity in 3 months (data not shown); therefore, retrospective analyses are challenging. Furthermore, nasal swab sample collection is inconvenient in live pigs as they have curved nasal cavities, and results can show large batch-to-batch variation [18, 21, 22]. Consequently, there remains an unmet need for a more sensitive and convenient serological method to detect *M. hyopneumoniae* infection in live naturally-infected pigs.

Evidence suggests there is a single strongly immunoreactive epitope on the Mhp366 protein that reacts with porcine sera raised by natural *M. hyopneumoniae* infection but not bacterin vaccination [11]. Our previous research shows that Mhp366 is recognized by IgG antibodies induced by *M. hyopneumoniae* infection, suggesting that Mhp366 represents a candidate antigen for a new IgG-ELISA [13]. The objective of the present study was to develop a *M. hyopneumoniae* convalescent serum IgG-ELISA using Mhp366 as the coating antigen for the detection of anti-*M. hyopneumoniae* IgG in naturally infected pathogen-induced convalescent sera under field conditions. Mhp366 is a 555 amino acid protein with a molecular weight of 64.4 kDa (calculated). As the epitope recognized by *M. hyopneumoniae* convalescent sera includes the amino acids 68–88 (^68^QKENSQKNDVVNSQNKTEKTE^88^), a 637 bp fragment of the *mhp366* gene that covers the differential diagnostic region was amplified.

Reproducibility was measured by determining intra- and inter-assay variation. The intra-assay CV ranged from 3.27 to 7.26 %, and the inter-assay CV ranged from 3.46 to 5.93 %. Based on these results, the proposed method has good reproducibility. In addition, our ELISA was able to differentiate anti-*M. hyopneumoniae* antibodies from antibodies against 7 other pathogens that cause porcine respiratory disease. Generally, sera applied in diagnostic ELISAs for porcine pathogens are diluted from 1:40 to 1:200. However, in our method, the optimum serum dilution was 1:500, and the maximum dilution was 1:2,000. These data suggest that our novel *M. hyopneumoniae* IgG ELISA requires a small amount of sample compared to other commercially available IgG-ELISAs, and is reproducible, specific, and sensitive.

Determination of a cut-off value is always a difficult step in the standardization of an ELISA. Multiple criteria may be used to determine a cut-off value, including ROC curve analysis or the mean of the negative control serum samples plus 2 standard deviations. The method chosen to determine a cut-off value should be considered in the context of the test that is under investigation. For ROC analysis, we could have used a commercial IgG-ELISA to screen clinically confirmed *M. hyopneumoniae* positive and negative sera. However, as we had abundant negative sera (n = 180 samples), we used the mean OD_450_ of the negative sera plus 3 standard deviations (SDs), and compared the results from 80 clinically confirmed *M. hyopneumoniae* positive and negative sera on the commercial IgG-ELISA and the convalescent serum IgG ELISA. The number of sera used to calculate the cut-off value by mean OD_450_ + 3SDs was larger than that available for ROC analysis, therefore, mean OD_450_ + 3SD was considered the best approach to calculate the cut-off value for our ELISA.

We compared our novel *M. hyopneumoniae* convalescent serum IgG-ELISA with the sIgA-ELISA, which is currently considered the most accurate method to detect natural *M. hyopneumoniae* infection. 96.3 % of the sera tested on both the sIgA-ELISA and convalescent serum IgG-ELISA gave consistent results, including 97.3 % of the sera that tested positive and 83.3 % of the sera that tested negative. Only 56.3 % of the sera tested on both the sIgA-ELISA and commercial IgG-ELISA gave consistent results, including 58.1 % of the sera that tested positive and 58.1 % of the sera that tested negative. These data suggest the accuracy of our *M. hyopneumoniae* convalescent serum IgG-ELISA was comparable to the sIgA-ELISA but better than the commercial IgG-ELISA. The *M. hyopneumoniae* convalescent serum IgG-ELISA detected no anti-*M. hyopneumoniae* IgG in hyperimmune serum samples, while a commercial IgG-ELISA identified 95/145 of these sera as positive. These data indicate that our novel ELISA can distinguish between natural infections and hyperimmune sera.

In the present study, the prevalence of *M. hyopneumoniae* DNA detected by nested PCR in laryngeal swabs was much lower than the prevalence of anti-*M. hyopneumoniae* antibody detected in sera by the IDEXX IgG-ELISA (57.5 %) and our *M. hyopneumoniae* convalescent serum IgG-ELISA (91.3 %), or in nasal swabs by the sIgA-ELISA (92.5 %.). The discrepancy in prevalence of *M. hyopneumoniae* detected by nested PCR and ELISA may be explained by the disparate sampling sites. *M. hyopneumoniae* may not be present in laryngeal swabs in all infected pigs, but all infected pigs will produce *M. hyopneumoniae* antibody, which may be detectable for several weeks after infection.

Some studies indicate that seroconversion in natural *M. hyopneumoniae* infection takes about 28 days [9, 23], while the mucosal response can be identified 6 days after infection [9, 24]. Our future studies will determine the earliest time point post-infection at which anti-*M. hyopneumoniae* IgG can be detected by our *M. hyopneumoniae* convalescent serum IgG-ELISA by testing a set of sera from experimentally infected pigs and pigs naturally infected but followed longitudinally where the establishment of infection can be proven and time of seroconversion established.

## Conclusions

In conclusion, we have developed a reproducible, specific, and sensitive indirect ELISA to detect anti-*M. hyopneumoniae* IgG in naturally infected pathogen-induced convalescent sera. This ELISA could be used to carry out large scale surveillance of *M. hyopneumoniae* infection in pig farms regardless of vaccination status.

## Methods

### Cloning of the*mhp366-N*gene fragment

The *mhp366-N* gene fragment was cloned as previously described [12]. Briefly, a HiPure Plasmid Micro Kit (Magen, China) was used to extract plasmid pGEX-6P-2-mhp366 from the recombinant bacterium GST-Mhp366 [12], according to the manufacturer’s instructions. The *mhp366-N* fragment containing the peptide segment that reacted with *M. hyopneumoniae* convalescent sera but not hyperimmune sera was amplified using PrimeSTAR® Max DNA Polymerase (Takara, China) and the following primers: 5’-CGCGGATCCATGAAAAAAATGGTAAAATATTTTCTAG-3’ (*Bam*H I) and 5’-CCGCTCGAGCCAAAATGGGCCACCGTT-3’ (*Xho* I). A recombinant plasmid was constructed by ligating the PCR product into the vector pET-28a(+). The ligation product was transformed into *E. coli* DH5α competent cells, and was verified using double restriction enzyme digestion and sequencing.

### Expression and purification of recombinant Mhp366-N protein

Recombinant Mhp366-N protein was expressed and purified. Briefly, *E. coli* BL21(DE3) competent cells were transformed with recombinant plasmid and cultured at 16℃ for 20 h in Luria-Bertani media containing 50 µg/mL kanamycin and 1 mM IPTG. Ni affinity chromatography (GE Healthcare, USA) (gradient of 0.1-1 M imidazole) was used to purify recombinant Mhp366-N protein, which was identified with SDS-PAGE and Western blot, and quantified with the BCA protein assay kit (Beyotime, China).

### Animals

Animal experiments were performed according to the Guide for the Care and Use of Laboratory Animals of the Ministry of Health, China. Ethical approval for this study was provided by the Institutional Animal Care and Use Committee of Southwest University (Approval No. IACUC-20170921-3). Written informed consent was obtained from all participating farm owners after they had received an explanation of the objectives, protocols, and potential risks associated with the study.

Three farms provided serum samples for use in this study. Pigs from Farm A had no PEP-like clinical syndromes or lung lesions and were *M. hyopneumoniae*-free based on bacterial culture and amplification of *M. hyopneumoniae* DNA by nested PCR. In addition, sera were *M. hyopneumoniae* IgG negative based on immunological diagnosis with a commercial ELISA kit (IDEXX laboratories, Westbrook, Maine, USA). These assays had been performed in the last 2 years. Pigs from Farm B and Farm C had a history of exposure to *M. hyopneumoniae* within the last 2 years according to clinical signs and serological surveillance, but had not been vaccinated against *M. hyopneumoniae*. Approximately one quarter of the pigs at Farm B had clinical signs of PEP. Whereas, PEP occurred sporadically among the pigs at Farm C. All pigs were weaned at day 21 after birth.

### Sample collection and preparation

At Farm A, sera were collected from the cranial vena cava of 180 unvaccinated pigs at week 10 after birth and on day 56 after the last immunization from 145 pigs that had been immunized with a commercial inactivated adjuvanted (mineral oil and aluminum hydroxide) whole cell *M. hyopneumoniae* vaccine (MYPRAVAC SUIS, Hipra Lab) on day 7 and day 21 after birth. Laryngeal swabs were obtained after restraining the pigs, as previously described [18]. At Farm B and Farm C, sera were collected from the cranial vena cava of 20 pigs at week 21 after birth and 80 pigs aged > 200 days, respectively. Laryngeal swabs were also collected from the pigs at Farm B [18], and nasal and laryngeal swabs were collected from the pigs at Farm C, as previously described [10]. After sample collection, the pigs were released.

Glycerol, to a final concentration of 50 %, was added to sera, which was stored at -20 °C in aliquots. Samples from laryngeal and nasal swabs were resuspended into 1 mL sterile PBS at 4℃ overnight and then centrifuged at 12,000 g for 10 min. *M. hyopneumoniae* DNA was detected in the supernatant of the laryngeal swabs using nested PCR, as previously described [10]. SIgA was identified in the supernatant of the nasal swabs using the sIgA-ELISA kit, according to the manufacturer’s instructions [10].

### Optimization of the*M. hyopneumoniae*convalescent serum IgG-ELISA

The *M. hyopneumoniae* convalescent serum IgG-ELISA was optimized. Briefly, the wells of microtiter plates (Corning Inc., USA) were coated with 100 µL 0.25 µg/mL to 8 µg/mL Mhp366-N protein in coating buffer (0.5 M carbonate buffer, pH 9.6) at 37℃ for 1 h and at 4℃ overnight. After discarding unbound antigen, the wells were washed five times with PBS containing 0.05 % Tween-20 (PBST). Non-specific binding was blocked with 200 µL PBST, 1 % BSA, 2.5 % skim milk, 10 % fetal bovine sera (FBS), 1 % gelatin, or 1 % ovalbumin at 37℃ for 0.5 h, 1 h, or 2 h. After washing with PBST, 100 µL serum (1:50 to 1:8,000) were added and incubated at 37℃ for 0.5 h, 1 h, or 2 h. After washing with PBST, 100 µL of HRP-conjugate rabbit anti-pig IgG (H + L) secondary antibody (Invitrogen, USA) (diluted from 1:10,000 to 1:80,000 in blocking buffer) were added and incubated at 37℃ for 0.5 h, 1 h, or 2 h. After washing with PBST, 50 µL of substrate A (100 mL H_2_O containing anhydrous sodium acetate 2.72 g, citric acid monohydrate 0.35 g, 30 % hydrogen peroxide 0.06 mL) and substrate B (100 mL H_2_O containing EDTA·Na_2_ 0.04 g, citric acid monohydrate 0.2078 g, glycerol 10 mL, TMB·2HCl 0.0391 g) were added, incubated at RT for 5 min, 10 min or 15 min, and the reaction was ended with 50 µL 2 M H_2_SO_4_. Optical density at 450 nm (OD_450_) was recorded with an ELISA plate reader (ThermoFisher Scientific, Ratastie 2, FI-01620 Vantaa, Finland). Each experiment was performed at least twice, and all samples were assayed in triplicate. Working conditions were optimized by determining the highest *M. hyopneumoniae* convalescent (P)-to-negative (N) sera OD_450_ ratios.

### Cut-off value for the*M. hyopneumoniae*convalescent serum IgG-ELISA

Descriptive statistics (mean and SD) were generated using SAS 8.2 statistical software (SAS Institute, Inc. Cary, NC, USA). The cut-off value was determined by calculating the mean OD_450_ of the negative sera from Farm A plus 3 standard deviations (SD), as previously described [25, 26].

### Reproducibility of the*M. hyopneumoniae*convalescent serum IgG-ELISA.

Intra- and inter- assay variation (coefficient of variation [CV]) between runs were evaluated as previously described [10]. Briefly, 2 *M. hyopneumoniae* negative and 2 convalescent sera were randomly selected. The negative sera were from Farm A and were negative on PCR. The convalescent sera were from Farm B, and were positive on PCR. Five replicates of each sample were run in one batch to evaluate intra-assay (within plate) variation and 3 plates were run as separate batches to evaluate inter-assay (between runs) variation.

### Specificity and sensitivity of the*M. hyopneumoniae*convalescent serum IgG-ELISA.

*M. hyorhinis* (Mhr), *A. pleuropneumoniae* (App), *S. suis* serotype 2 (SS2), classical swine fever virus (CSFV), porcine reproductive and respiratory syndrome virus (PRRSV), porcine circovirus type 2 (PCV2) and pseudorabies virus gB protein (gB-PRV) positive sera that were detected using an established ELISA, and 2 *M. hyopneumoniae* negative and 2 convalescent sera that served as negative and positive controls, respectively, were used to determine specificity.

Five *M. hyopneumoniae* convalescent sera diluted at 1:100, 1:500, 1:1,000, 1:2,000, 1:4,000, 1:8,000, 1:16,000, and 1:32,000 in blocking buffer were tested using the other optimized working conditions of the ELISA. The sensitivity of the ELISA was evaluated based on the cut-off.

### Comparison of the*M. hyopneumoniae*convalescent serum IgG-ELISA with commercial kits.

Anti-*M. hyopneumoniae* IgG in sera from vaccinated pigs at Farm A and unvaccinated pigs at Farm C was detected with the commercial IDEXX IgG-ELISA kit and the *M. hyopneumoniae* convalescent serum IgG-ELISA. SIgA in nasal swabs collected from Farm C was detected with the sIgA-ELISA kit, kindly provided by Dr. Zhixin Feng. *M. hyopneumoniae* DNA in laryngeal swabs was detected using nested PCR [10]. Each sample was assayed in duplicate.

### SDS-PAGE and western blot

Pretreated bacteria or purified protein were solubilized and loaded onto SDS polyacrylamide gels. After electrophoresis, samples were stained with Coomassie brilliant blue, or transferred (2 h at 100 V) to a polyvinylidene difluoride membrane (Roche Diagnostics, German) using a transblotting apparatus (Bio-Rad, USA). The membrane was blocked in 5 % skimmed milk-TBST at 4℃ overnight. The membrane was incubated with His-tag (4C2) monoclonal antibody (1:8,000; Bioworld Technology, China) at RT for 1 h followed by horseradish peroxidase (HRP)-conjugated goat anti-mouse IgG (1:20,000; Proteintech, China) at RT for 1 h, and visualized with enhanced chemiluminescence (CWBio, China).

## Data Availability

The dataset analyzed during the current study is available from the corresponding author on reasonable request.

## Abbreviations

CV: Coefficient of variation
ELISA: Enzyme-linked immunosorbent assay
HRP: Horseradish peroxidase
OD: Optical density
PBS: Phosphate buffer solution
PEP: Porcine enzootic pneumonia
PRDC: Porcine respiratory disease complex
SD: Standard deviations
